# 国产电磁导航支气管镜系统引导下诊断、定位和治疗技术规范专家共识(2021版)

**DOI:** 10.3779/j.issn.1009-3419.2021.101.30

**Published:** 2021-08-20

**Authors:** 中国医学装备协会呼吸病学装备技术专业委员会, 国产电磁导航支气管镜技术专家组

**Keywords:** 电磁导航支气管镜, 肺外周病变, 诊断, 定位, 治疗, Electromagnetic navigation bronchoscopy, Peripheral pulmonary lesion, Diagnosis, Localization, Treatment

## Abstract

电磁导航支气管镜(electromagnetic navigation bronchoscopy, ENB)是以电磁定位技术为基础，结合虚拟支气管镜、三维计算机断层扫描(computed tomography, CT)成像和呼吸门控技术的新型支气管镜检查手段，该技术已在临床中广泛应用。近年来，国产电磁导航系统也得到了较快发展，其在肺外周病变诊断、定位和治疗中的有效性和安全性已得到初步验证，为优化和标准化国产ENB技术操作规范，指导其在临床实践中的应用，中国医学装备协会呼吸病学装备技术专业委员会和国产电磁导航支气管镜技术专家组组织专家讨论并制定了本专家共识。

## Abstract (en)

Electromagnetic navigation bronchoscopy (ENB) is a novel type of bronchoscopy based on electromagnetic positioning technique combined with virtual bronchoscopy, three-dimensional computed tomography (CT) imaging and respiratory gating technique, which has been widely applied in clinic practice. In recent years, the domestic electromagnetic navigation system has also been developed rapidly, and its effectiveness and safety in the diagnosis, localization, and treatment of peripheral pulmonary lesions have been initially verified. In order to optimize and standardize the technical specifications of domestic ENB and guide its application in clinical practice, the consensus statement has been organized and written in a collaborative effort by the Professional Committee on Respiratory Equipment Technology of Chinese Medical Equipment Association and the Expert Group on Technical of Domestic Electromagnetic Navigation Bronchoscopy.

## 1 引言

电磁导航支气管镜(electromagnetic navigation bronchoscopy, ENB)是以电磁定位技术为基础，结合虚拟支气管镜、三维计算机断层扫描(computed tomography, CT)成像和呼吸门控技术的新型支气管镜检查手段。自1998年开展动物实验，2003年开展首例人体临床研究，现已被国内外相关指南推荐为肺外周病变(peripheral pulmonary lesion, PPL)经支气管肺活检术(transbronchial lung biopsy, TBLB)的诊断工具，近年来临床应用日益广泛，已拓展至ENB引导下行外科术前定位肺结节和治疗肺部肿瘤^[1-4]^。现获中国或美国食品药品监督管理局认证的ENB系统分别为中国朗开公司研发的LungCare系统、美国Medtronic公司研发的superDimension系统和美国Veran公司研发的SPiN系统。三者均具有各自的特点，LungCare配有不同型号的定位导线(locatable wire, LW)，兼容全系列支气管镜和不同规格的引导鞘管(guided sheath, GS); superDimension需搭配工作通道内径2.6 mm以上的治疗支气管镜，通过旋转操纵杆控制预弯鞘管和LW的方向; SPiN则集成经支气管和经胸壁两种导航路径模式，且取材工具头端搭载传感器，可实时跟踪工具位置。

立足于国家重点研发计划“基于国产电磁导航系统的早期肺癌精准诊疗技术集成解决方案研究(No.2017YFC0112700)”项目，国产LungCare电磁导航系统已推广至国内多家医院，开展系列大样本临床研究，验证其在诊断、定位和治疗中的有效性和安全性。为优化和标准化国产ENB技术操作规范，指导其在临床实践中的应用，特制定本专家共识。因现阶段尚缺乏关于国产ENB临床应用的高级别循证医学证据，故本共识是在临床实践经验的基础上，结合国产ENB临床研究文献和会议发言，并参考其他相关指南和共识，经讨论和综述而成，仅代表由中国医学装备协会呼吸病学装备技术专业委员会牵头的国产ENB技术专家组对一般建议的一致意见，不设证据类别和推荐等级^[3-20]^。随着以后更多国产ENB系统的研发上市，本共识将进一步更新。

## 2 适应证和禁忌证

### 2.1 适应证

ENB诊断技术的适应证：①直径 > 8 mm，性质不明，需行病理学或病原学诊断的PPL，尤其适用于常规支气管镜难以诊断的PPL; ②对已明确性质的PPL，治疗过程中根据病情需要对病变进行再次活检，指导后续治疗。PPL定义为影像学表现发生在段支气管远端被肺实质所包绕的肺部阴影，且常规支气管镜检查不可见的病变，无支气管腔内病变、黏膜下浸润等^[21, 22]^。

ENB定位技术的适应证：直径≤2 cm，倾向恶性，拟行胸腔镜下亚肺叶切除术，术者评估术中直视或触摸定位困难需行术前辅助定位的磨玻璃肺结节。

ENB治疗技术的适应证：鉴于目前ENB治疗技术主要是肿瘤消融治疗，因此本共识仅限于该适应证。根治性消融是针对术前经病理学和影像学检查明确为肿瘤最大径≤3 cm，且无其他部位转移的病灶，包括不适合手术或拒绝手术的初治或复治后局部进展的周围型肺癌，以及单肺病灶数≤3个、双肺病灶数≤5个的肺转移瘤。姑息性消融其适应证可相对放宽，如病灶大小和数量超过根治性消融标准者。

### 2.2 禁忌证

本禁忌证与常规支气管镜检查禁忌证相同，具体内容参见成人诊断性可弯曲支气管镜检查术应用指南(2019年版)^[13]^。碘过敏者不宜行如吲哚菁绿(indocyanine green, ICG)等含碘剂定位。安装心脏起搏器患者，不宜行射频消融。

## 3 设备和器械

### 3.1 ENB系统

包括肺E助手、医学影像工作站设备、专用检查床、体位探测器、LW和信号采集器^[5, 6]^。肺E助手：集DICOM数据传输、支气管/血管/胸膜分割重建、导航路径规划、虚拟内窥等功能于一体的数据后处理云平台管理系统。医学影像工作站设备：控制信号的接收和处理，术中提供实时导航影像显示，如传感器和预设目标的三维坐标及方向信息，并提供前进路线图指示。专用检查床：内嵌磁场发生器产生均匀稳定的磁场，配合X光设备时转动手摇轮移动磁场发生器。体位探测器：三个体位探测器头端分别贴于胸骨柄、第八肋骨和左右腋前线交叉点附近，且需保证在有效磁场范围内，捕获呼吸运动。LW：一般和GS搭配使用，有外径0.75 mm、1.15 mm、1.45 mm和1.95 mm四种规格，术中通过LW头端的传感器实时提供其位于支气管树中的空间位置。信号采集器：连接体位探测器和LW的尾端，收集信号。

### 3.2 支气管镜

国产ENB系统可和所有类型的支气管镜配合使用，常用的包括细支气管镜(例如BF-P290，Olympus; 先端部外径4.2 mm，工作通道内径2.0 mm)和治疗支气管镜(例如BF-1TQ290，Olympus; 先端部外径5.9 mm，工作通道内径3.0 mm)。

### 3.3 支气管内超声(endobronchial ultrasound, EBUS)

EBUS为非嵌入式深入支气管管腔内，围绕超声换能器作圆周环形扫描，得到垂直于超声探头的横断面图像，可观察管腔周围组织结构情况，了解病灶和支气管的关系。常用外径1.4 mm、1.7 mm的EBUS探头(例如UM-S20-17S、UM-S20-20R，Olympus)。

### 3.4 引导鞘管套装

包括GS、活检钳和细胞刷等，其中GS和LW搭配，以建立工作通道，便于后续操作。例如常用来和外径1.45 mm、1.95 mm LW配合使用的GS套装(K201、K203，Olympus)，内含外径1.95 mm、2.55 mm的GS、外径1.5 mm、1.9 mm的活检钳和外径1.4 mm、1.8 mm的细胞刷。

### 3.5 放射影像设备

常用放射影像设备为具有透视功能的移动式C型臂，有条件者可购置具有三维成像功能的锥形束CT(cone-beam CT, CBCT)。

### 3.6 定位相关药物和耗材

常用生物染料有亚甲蓝和ICG，金属标记有弹簧圈等。行ICG定位时，应配备近红外荧光胸腔镜。行金属标记定位时，应配备放射影像设备。

### 3.7 治疗相关器械

现有相对成熟的经支气管消融治疗器械有柔性微波消融针和射频消融针。

## 4 人员配置

ENB技术属于三四级呼吸内镜诊疗技术，应由接受过系统培训、具有丰富经验的术者和助手操作或在其指导下完成。对于行全身麻醉的患者，配备麻醉医护人员。

## 5 术前评估和管理

详细询问患者现病史、过敏史，完善术前相关血液学检验和心肺功能、影像学检查，排查支气管镜检查相关禁忌证。进行层厚0.5 mm-1 mm、层间距0.5 mm-1 mm、分辨率512×512像素的薄层胸部增强CT扫描，了解靶病灶位置、性质、大小，以及和周围血管、脏器等组织结构的关系。

为降低出血风险，术前应停用抗凝和抗血小板药物，以及抗血管生成类药物，相关内容参见成人诊断性可弯曲支气管镜检查术应用指南(2019年版)。

通过健康宣教和心理疏导减轻患者焦虑紧张情绪，嘱予呼吸锻炼，以加强术中配合，提高对检查的耐受程度。

## 6 术前告知和知情同意

术前应详细告知患者本人及其委托人现有和替代诊疗方案，以及关于ENB诊疗技术的目的、利弊，可能存在的风险及处理方法等事项，签署书面知情同意书。

## 7 麻醉和气道管理

术前常规测量血压，心电监护，建立静脉通路。ENB操作可在联合或不联合镇静的局麻或全麻下进行，依据患者意愿和不同术式选择麻醉方式，以及合适的气道管理方式，一般推荐喉罩或8^#^气管插管。当进行外科手术时，行气管插管加用阻塞导管或双腔气管插管。

## 8 术前准备

### 8.1 术前计划

阅读患者胸部影像学资料，规划最佳的术前导航路线图，制定诊疗计划，如联合引导支气管镜技术方式、定位位点和消融治疗参数等。将DICOM格式的CT数据导入电磁导航规划软件，进行三维重建生成虚拟支气管树图像，勾勒靶病灶形态，标记靶病灶所在位置，优先选择通向靶病灶的目标支气管，设定导航路径。

### 8.2 设备和器械准备

选择合适的引导支气管镜技术，如支气管镜型号，是否联用EBUS-GS或透视等，并进行相关准备工作。按诊疗需求，分别用锁止扣、超声(ultrasound, US)卡锁和元器件(element tools, ET)卡锁标记LW、EBUS、细胞刷、活检钳和引导鞘管的相对位置，确保伸出鞘管的LW头端、EBUS换能器、活检钳钳杯三者相平齐，而细胞刷和引导鞘管的出口相平齐^[5]^。

## 9 术中操作

### 9.1 ENB技术操作规范

① 患者平卧于专用检查床上，麻醉后常规行白光支气管镜检查，清理气道分泌物，观察管腔和黏膜情况。将带有LW的GS插入支气管镜工作通道，使LW头端伸出工作通道少许，置于隆突正上方，分别走行于左右总支至下叶支气管和气管进行电磁位置信息的注册配准。配准结束后，支气管树呈绿色，查看匹配度，如若匹配度低于80%，可重新进行注册配准; ②注册配准结束后，再次将支气管镜置于隆突处，待3个呼吸曲线后再行电磁导航，同时观察镜下实时图像和虚拟内窥图像的吻合度，如若图像偏差太大，可以在支气管镜能够到达的最远端支气管内设置校正点，从而校正导航期间潜在的偏差; ③术中依据电磁导航实时引导的路径，尽可能地推进支气管镜至外周支气管，当不能继续前进时，通过调整支气管镜螺旋和(或)旋转环，控制GS行进方向，将带有LW的GS逐级深入各级支气管，向靶病灶所在位置推进; ④ENB系统可从不同三维层面实时跟踪LW位于支气管树的空间位置，显示与靶病灶中心点的距离，当ENB系统提示LW到达靶病灶后，由助手固定支气管镜，撤出LW，留置GS，利用GS建立延长工作通道，根据需要放置器械完成后续诊断、定位和治疗等操作。

### 9.2 诊断技术操作规范

以下以最常见的ENB联合应用EBUS-GS引导下的TBLB即ENB-EBUS-GS-TBLB技术为例，说明诊断技术操作流程。①沿上述留置的GS插入EBUS探头进行超声扫描确认病灶位置，可前后微调EBUS-GS位置以显示病灶内部结构获取满意EBUS图像，并观察病灶与支气管的关系(无/通向/邻近)，病灶的形态、近端、远端和最大截面，以及是否邻近血管等; ②回撤GS至距病灶最大截面1 cm左右，或至病灶近端，并用ET卡锁标记GS的位置(即Stopper点，距工作通道入口约1 cm)，移出EBUS探头，留置GS在原位，随后通过GS进行刷检和活检，送检细胞学和组织学检查，必要时送检微生物学检查; ③刷检时注意控制刷头出鞘管的长度。活检时手持GS的Stopper点上缘向前推进，确保活检钳完全张开，到位后关闭钳杯进行活检，撤出活检钳时，依然固定GS的Stopper点上缘，避免GS滑动和变形。重复活检时，GS向后回撤至初始标记位置，或根据取材的情况适当调整位置; ④活检结束后，可留置GS 1 min左右以预防性压迫止血。如观察到明显出血，局部给予冰生理盐水、止血药物等处理，严重者可给予球囊压迫、介入等综合治疗; ⑤如需联合透视辅助，常规应用方法如下：术前依据胸部CT横断面病灶所在位置将其投射于胸部CT的预扫描正位片中，依据重要解剖结构(如肋骨、主肺动脉等)初步判断其大致位置。操作前，依据上述位置信息预先行透视定位照，再次确认其位置。对于小病灶和磨玻璃病变等透视不可见情况，更加依赖术前胸部CT投射定位图; 对于受心脏、膈肌遮挡的病灶，可通过调整C形臂的角度来暴露病灶。术中操作时，可于透视下确认LW是否到位，必要时根据透视调整LW的方向和位置。到达病灶后，留取EBUS探及病灶最大层面的透视定位照为参考图，随后在透视监视下取材。

### 9.3 定位技术操作规范

① 生物染料定位法：沿上述留置的GS注入稀释后的亚甲蓝或ICG 0.5 mL-1 mL，再沿GS注入20 mL左右空气，留置GS 1 min左右后撤出，观察管腔内生物染料返流情况。金属标记定位法：将金属标记推送至上述留置的GS内，利用搭配的输送导丝或活检钳等工具将其完全推送出GS，随后撤出GS; ②定位结束后，将定位位点投射于虚拟肺图中，模拟肺膨胀状态下其位于胸膜表面的投射标记点。患者转运至手术床上，取健侧卧位，全身麻醉、单肺通气，行胸腔镜下楔形或肺段亚肺叶切除术; ③生物染料定位法：应用普通或近红外荧光胸腔镜直视观察亚甲蓝或ICG在脏层胸膜的分布情况，依据染色范围指导手术切除范围。金属标记定位法：依据触摸或透视下金属标记的位置，用卵圆钳夹闭拟切除的肺组织边缘; ④在成功切除肺组织标本后，立即解剖确认靶病灶位于其内，观察生物染料染色区域或金属标记和靶病灶的位置关系，评估定位的准确性。如若未能成功切除靶病灶，应扩大手术切除范围或更改术式行肺叶切除术。

### 9.4 治疗技术操作规范

EBUS-GS操作部分详见诊断技术操作规范。选取通向靶病灶中心的目标支气管，标记EBUS探头位于病灶不同位置的透视定位照，在透视监视下将消融针(可不联用GS)插入到靶病灶进行消融治疗。消融范围应包括靶病灶及瘤周5 mm-10 mm的正常肺组织，以达到完全消融目的。

## 10 术后处理和随访

对于病理学恶性、特异性良性病理证据、病原学阳性的患者，作后续进一步治疗; 对于非特异性良性病理证据、病原学阴性的患者，结合临床及影像学表现，可给予经验性治疗和定期随访，如病理学结果和影像学表现不一致时，可行再次活检或手术; 对于获取的标本不合格，未能明确诊断的患者，建议再次活检、手术或定期随访。

对于ENB引导下的消融治疗，术后1 d应行胸部平扫CT或胸片观察消融范围判断消融技术的成功率和并发症情况。推荐使用改良的实体瘤疗效评价标准进行疗效评价，胸部增强CT是目前评价消融治疗效果的常用方法，有条件者可结合正电子发射型计算机断层显像(positron emission computed tomography, PET)-CT和磁共振成像(magnetic resonance imaging, MRI)。

## 11 常见并发症

相对于经胸壁肺穿刺技术，ENB技术经自然腔道进行，不损伤肺部正常组织结构，气胸、出血等并发症明显降低^[20-22]^。迄今为止，最大样本量关于ENB诊治技术的NAVIGATE研究结果^[23]^显示，最常见的并发症为气胸(2.9%)和出血(1.5%)。

在ENB诊断技术方面，几项荟萃分析^[23-25]^统计了诊断相关并发症，气胸平均发生率为3.1%-5.9%，其中2%需行胸腔闭式引流，出血平均发生率为0.9%-1.8%，其他并发症包括呼吸衰竭、胸痛、发热等。

在ENB定位技术方面，一项荟萃分析^[26]^提示，ENB引导下定位所致胸膜损伤和出血的发生率分别为0.03(95%CI: 0.01-0.06)和0.00(95%CI: 0.00-0.00)。此外，两项小样本、回顾性比较CT和ENB两种引导方式下术前定位的临床研究^[27, 28]^也同样显示ENB定位技术的高安全性。

在ENB治疗技术方面，经支气管消融治疗目前正进行探索性临床研究阶段，其并发症和经皮消融技术类似，主要表现为气胸、出血、胸痛、发热、胸腔积液等^[29]^。

综上，由于经自然腔道进行，ENB诊治技术总体是较为安全的，但术后仍应密切观察。并发症与电磁导航技术无关，主要与诊断、定位和治疗操作有关，另治疗技术的风险相对高于诊断和定位技术。

## 12 临床应用推荐

经支气管诊治的难点在于如何在复杂的支气管迷路中发现通往靶病灶的最佳路径，快速寻找到目标病灶，并确保器械准确到达病灶中。近年来以ENB为代表的引导支气管镜技术在临床上应用逐渐普及，ENB可通过实时导航准确定位，缩短寻找病灶的时间，提高技术成功率，是解决上述难点的有效手段^[1]^。众多临床研究已明确了ENB在诊断PPL中的临床应用价值，其还可以进行术前定位为微创胸外科精准局部手术切除提供指导，以及引导进行经支气管消融治疗肺部肿瘤，大大拓展了对PPL的经支气管诊疗方式，提供了一种安全有效的新技术。基于现有文献总结和临床实践经验，本共识作出以下推荐意见：

### 12.1 ENB引导下的诊断技术推荐

#### 12.1.1 推荐常规使用细支气管镜

相对于治疗和标准支气管镜而言，细支气管镜可到达更远端支气管从而更好辨认目标支气管; 相对于超细支气管镜而言，细支气管镜可结合现有的EBUS-GS技术使用，从而平衡外周可达性、取材能力和操作的便利性。

#### 12.1.2 推荐常规联合应用EBUS-GS

EBUS-GS的技术优势包括^[30-33]^：①确认到达病灶，判断支气管走行和病灶的关系，并显示病灶内部结构，包括血管、钙化、坏死等，预测良恶性性质; ②固定引导鞘管和病灶的相对轴线位置，间接保证通过引导鞘管进出的器械位于病灶内部，从而无需再次寻找和确认病灶，确保取材位置的一致性，减少取样时间; ③一定程度上可以利用GS压迫止血，降低因反复多次取材导致的出血; ④操作相对简便，无辐射风险。但EBUS技术对磨玻璃病变可视性差，严重依赖术者经验^[31]^。一项前瞻性随机对照试验^[34]^表明，ENB和EBUS联合诊断率(88%)明显高于单独EBUS(69%)和单独ENB(59%)的诊断率，EBUS和ENB联合应用可以克服每种技术的局限性，可在不影响安全性的情况下提高对PPL的诊断率。ENB是寻找并确认到达病灶的有力工具，而EBUS可将病灶可视化，明确病灶的具体位置，以及和支气管的结构关系，优先选择通向靶病灶的目标支气管进行活检，两者结合提高对病灶的诊断效能^[34-37]^。

#### 12.1.3 推荐必要时联合应用透视

透视的价值主要体现在：观察呼吸运动对病灶位置的影响(尤其下叶病灶)，引导(避开细支气管分叉)和确认器械准确到达病灶，标记器械和病灶的相对空间位置，监视取样过程，确认活检钳开闭状态及细胞刷刷检范围，确保器械不偏离病灶所在位置，同时避免因呼吸运动、小病灶活检或其他情况导致的移位^[31, 38]^。一项前瞻性随机对照研究^[39]^表明，对平均直径27.65 mm的PPL在应用VBN和EBUS的前提下，联合或不联合透视的诊断率虽无统计学意义，但联合组的诊断率稍高于不联合组，尤其在 < 2 cm病变中，诊断率差异明显增大。美国胸科医师学会指南表明，ENB引导的TBLB可以在有无透视引导下进行，是对EBUS-GS技术的补充。因此，常规ENB引导下的诊断无需透视辅助，但对于小病灶、以磨玻璃为主的病变、以及冷冻活检或针吸活检时，建议透视或CBCT辅助。

#### 12.1.4 推荐选择合适的取材方式和次数

取材方式有钳夹活检、冷冻活检、刷检、针吸活检、灌洗多种取材方式，以获取组织学标本为主，细胞学可作为有效补充，联合应用多种取材方式可增加诊断率^[14, 25]^。美国胸科医师学会指南提出，应基于病灶的大小、位置、与支气管的关系，以及风险，综合考虑选择取材方式^[40]^。如当EBUS图像显示为偏心性病变时，针吸活检有可能提高诊断率^[41]^。而对以磨玻璃为主的PPL，可以应用冷冻活检^[7]^。活检次数的增加与并发症的发生率相关，在考虑风险/获益比的情况下，推荐钳夹活检肉眼可见标本至少5块^[36, 42]^。

#### 12.1.5 推荐必要时联合应用快速现场细胞学评估

快速现场细胞学评估(rapid on-site evaluation, ROSE)是一种伴随于取材过程的即时细胞学判读技术，可判断取材的合格性，评估标本的充分性，指导介入方法，形成初步诊断或缩小鉴别诊断的范围，优化标本下一步的处理方案，已逐渐发展成为PPL诊断技术的重要辅助手段之一^[43, 44]^。研究^[45]^显示ROSE可提高一些疑难病例的诊断率，如位于尖段、最大径 < 3 cm、无CT支气管征的PPL。因此，在有条件的单位，对于诊断困难的PPL，推荐联合应用ROSE。

### 12.2 ENB引导下的定位技术推荐

#### 12.2.1 推荐常规应用ENB单一引导方式

术前对靶病灶精准定位有助于术中识别病灶的位置，指导切除范围，是提高胸腔镜下手术切除成功率和安全性的关键^[26, 46-48]^。两项小样本、回顾性的临床研究^[27, 28]^比较了CT和ENB两种引导定位技术的优缺点，结果显示ENB引导下的定位具有极高的安全性，且具有不弱于CT引导定位的有效性，是一种切实可行的定位方法。另有*meta*分析^[26]^提示ENB引导下有效定位的成功率为0.96(95%CI: 0.95-0.99)。虽然联合应用CT或透视等放射影像技术可进一步提高定位的精准性，但增加操作步骤和时间，以及辐射暴露风险。因此，一般推荐应用单一ENB引导下的定位技术^[8, 10]^。

#### 12.2.2 推荐吲哚菁绿行生物染料定位法

生物染料定位法是基于脏层胸膜染色范围定位靶病灶，可视性好，便于直视观察，更有利于术中寻找靶病灶，但其缺点主要是染料易于扩散性，定位后须尽早手术^[46]^。生物染料的注入量应与距胸膜的距离成正比，在显影性和弥散范围中间得到一个较好的平衡点，而对于位于前胸壁侧的病变，建议增加液体和空气注入量^[10]^。金属标记定位法是依靠标记物的膨胀性固定于支气管管腔内，不易移位，可长期放置无需立即手术，但其缺点主要是其术中需结合透视确定标记位置，具有辐射性^[46, 49]^。综上，一般推荐生物染料定位法。

生物染料主要包括亚甲蓝和ICG，前者在色素沉积的胸膜表面不易于观察，并且干扰切除后病灶的识别^[46, 48]^;后者依靠荧光激发显色，显影明亮清晰，不覆盖肺组织原有色彩，更利于观察。因此，推荐首选吲哚菁绿。

#### 12.2.3 ENB引导下的定位技术具体应用方法

ENB定位技术应结合手术方式和切除范围进行定位方法、位置和点数的选择，同时ENB定位技术不同于诊断技术，不受限于病灶与支气管的关系。对于肺外周1/3的病灶，于临近靶病灶的目标支气管进行单点定位即可。对于肺内周2/3的病灶，可分别于胸膜下和靶病灶处进行两点定位^[50, 51]^。当要求精确指导手术切缘、切除的深度等范围，则需行多点、多次三维立体定位，生物染料和金属标记联合定位尤佳^[52-54]^。

### 12.3 ENB引导下的治疗技术推荐

经支气管消融的难点在于确认消融针位于病灶的三维空间位置，以及消融术中疗效的监测，故常规推荐联合应用放射影像技术，尤其推荐CBCT或CT，以便于术中更精确地监测消融范围^[55, 56]^。其次，根据靶病灶性质、大小、位置和形状，选择消融治疗方式，调整消融功率、时间和点数等参数，尽可能选择具有同心圆型EBUS典型特征图像的目标支气管，对于有多条支气管通向或较大的靶病灶，建议行多条入路和多点消融，以确保足够的能量覆盖^[57]^。总之，ENB引导下的消融治疗仍处于探索阶段，虽有ENB等多种技术辅助定位，但仍需进一步研究术中精准导航、实时定位技术，建立完备的术中实时疗效评价体系^[58-60]^。

## 13 总结

国产ENB系统目前已广泛应用于临床中，基于现有实践经验和相关临床研究，特制定本共识。针对现有临床诊疗中的难题，基于ENB术前路径规划和术中实时导航功能，ENB可以通过带有电磁定位信息的探头快速准确到达靶病灶处，从而进行有效诊断、定位和治疗。ENB引导下的诊断技术，尤其适用于≤3 cm常规支气管镜难以到达的PPL，常规推荐进行ENB-EBUS-GS-TBLB术式，必要时可联用透视辅助。ENB引导下的定位技术，辅助肺结节胸腔镜术前定位，指导术者在术中快速、准确的定位目标病灶，以进行精准手术切除，常规推荐生物染料定位法。ENB引导下的治疗技术，主要适用于不适合手术和拒绝手术的早期周围型肺癌患者，可提供一种安全、有效、微创的局部治疗手段，常规推荐放射影像技术辅助，监测消融针位于病灶的空间位置，以及判断消融范围和效果。

**Table:** 国产电磁导航支气管镜技术专家组成员

	医院	姓名
执笔人	上海市胸科医院/上海交通大学附属胸科医院	陈军祥
执笔人	广州医科大学附属第一医院	陈小波
执笔人	上海市胸科医院/上海交通大学附属胸科医院	谢芳芳
执笔人	上海市胸科医院/上海交通大学附属胸科医院	孙加源
参与专家	东南大学附属中大医院	丁明
参与专家	中日友好医院	侯刚
参与专家	苏州大学附属独墅湖医院	蒋军红
参与专家	贵州航天医院	蒋婷
参与专家	广州医科大学附属第一医院	李时悦
参与专家	蚌埠医学院第一附属医院	李伟
参与专家	中国科学院大学深圳医院	龙发
参与专家	四川大学华西医院	刘丹
参与专家	嘉兴市第一医院	陶峰
参与专家	山东省公共卫生临床中心	王晓平
参与专家	中南大学湘雅二医院	肖奎
参与专家	贵州省人民医院	叶贤伟
参与专家	新疆医科大学第八附属医院	杨俊勇
参与专家	西安交通大学第二附属医院	杨拴盈
参与专家	山西省太钢总医院	杨鹏
参与专家	郑州大学附属第一医院	姚孟英
参与专家	四川省医学科学院/四川省人民医院	邹俊
参与专家	应急总医院	张楠
参与专家	海军军医大学第一附属医院	张伟
